# Electric Field‐Driven Conformational Changes in Molecular Memristor and Synaptic Behavior

**DOI:** 10.1002/advs.202505016

**Published:** 2025-04-30

**Authors:** Chanjin Lim, Taegil Kim, YoungJu Park, Daeho Kim, ChaeHo Shin, Suji Ha, Jin‐Liang Lin, Yuan Li, Junwoo Park

**Affiliations:** ^1^ Department of Chemistry Sogang University Seoul 04107 Republic of Korea; ^2^ Bruker Nano Surface Bruker Korea Co, Ltd. Seoul 05840 Republic of Korea; ^3^ Division of Chemical and Material Metrology Korea Research Institute of Standards and Science Daejeon 34113 Republic of Korea; ^4^ Key Laboratory of Organic Optoelectronics and Molecular Engineering Department of Chemistry Tsinghua University Beijing 100084 China; ^5^ Center for Nano Materials Sogang University Seoul 04107 Republic of Korea

**Keywords:** anion reaction dynamics, molecular electronics, molecular memristor, neuromorphic computing, quantum tunneling

## Abstract

This paper demonstrates the use of molecular artificial synapses in neuromorphic computing systems designed for low energy consumption. A molecular junction, based on self‐assembled monolayers (SAMs) of alkanethiolates terminated with *2*,*2*′‐bipyridine complexed with cobalt chloride, exhibits synaptic behaviors with an energy consumption of 8.0 pJ µm^−2^. Conductance can be modulated simply by applying pulses in the incoherent charge transport (CT) regime. Charge injection in this regime allows molecules to overcome the low energy barrier for C─C bond rotations, resulting in conformational changes in the SAMs. The reversible potentiation/depression process of conductance achieves 90% accuracy in recognizing patterns from the Modified National Institute of Standards and Technology (MNIST) handwritten digit database. The molecular junction further exhibits both rectifying and conductance hysteresis behaviors, showing potential for use in selector‐free synaptic arrays that efficiently suppress sneak currents.

## Graphical abstract

Self‐assembled monolayers (SAMs) based molecular device is used for the emulation of synaptic functions. The device operates with a novel switching mechanism via incoherent charge transport, inducing conformational changes. With a low energy consumption (8.0 pJ µm^−2^), the system shows promise for neuromorphic computing, offering potential in low‐energy, high‐efficiency applications.

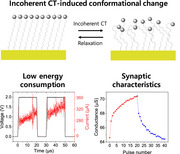

## 1 Introduction

With the rapid development of next‐generation technologies (e.g., artificial intelligence, self‐driving cars, and cryptocurrency), the speed and energy required for computation are increasing significantly.^[^ ^1^ ^]^ A neuromorphic system based on neural networks has seen drastic improvements in computation speed, overcoming the limitations of the von Neumann architecture. Approaches based on solid‐state electronics (e.g., ion migration, ferroelectricity, spintronics) have demonstrated the potential for artificial synapses, but they have not yet significantly reduced energy consumption and still generate heat. As the demand for energy‐efficient computing continues to grow, extensive research efforts have been devoted to developing low‐power device technologies.^[^ ^2^ ^]^ Molecular electronics, on the other hand, offer the promise of lower energy requirements due to the small electric currents and low energy barriers involved.^[^ ^3^ ^]^ In this work, we developed a molecular synaptic junction using SAMs of alkanethiolates terminated with chelates (*2*,*2*′‐bipyridine bound to cobalt chloride) between a gold electrode and eutectic gallium indium (EGaIn) liquid metal alloy, referred to as BIPY‐CoCl_2_ junctions. With a thickness of ≈2 nm, the BIPY‐CoCl_2_ junctions offer size advantages over thin‐film devices. The *I‐*‐*V* curve reveals conductance hysteresis due to conformational changes, indicating memristive characteristics. We presume that the injection of energetic electrons into the lowest unoccupied molecular orbital (LUMO) excites the molecule to a transient negative ion state, where increased vibration helps overcome the energy barrier for C─C bond rotation (e.g., 16–19 kJ mol^−1^ for butane),^[^ ^4^ ^]^ facilitating conformational changes. We validate our interpretation based on experimental results from confocal Raman spectroscopy and atomic force microscopy. The molecular junctions effectively emulate various synaptic characteristics such as paired‐pulse facilitation (PPF), spike‐amplitude dependent plasticity (SADP), and spike‐duration dependent plasticity (SDDP), and exhibit reversible potentiation/depression cycles under optimal pulses. We used gradual conductance modulation to simulate the recognition of MNIST patterns. The molecular junctions also display rectifying behavior in the *I‐*‐*V* curve, which efficiently suppresses sneak currents in the crossbar array.^[^ ^5^ ^]^ We demonstrated that the maximum crossbar array size can reach 73211 with a read margin of 10%. The BIPY‐CoCl_2_ junctions show potential as energy‐efficient molecular synaptic devices that can be fabricated into selector‐free crossbar arrays. The broad diversity of organic molecules allows for a wide range of electrical, optical, and chemical properties to be engineered into molecular junctions, enabling their use in various applications.^[^ ^6^ ^]^ Furthermore, the ease of synthesizing molecules with specific properties allows the optimization of molecular junctions for further applications.

## 2 Results and Discussion

**Figure 1:**
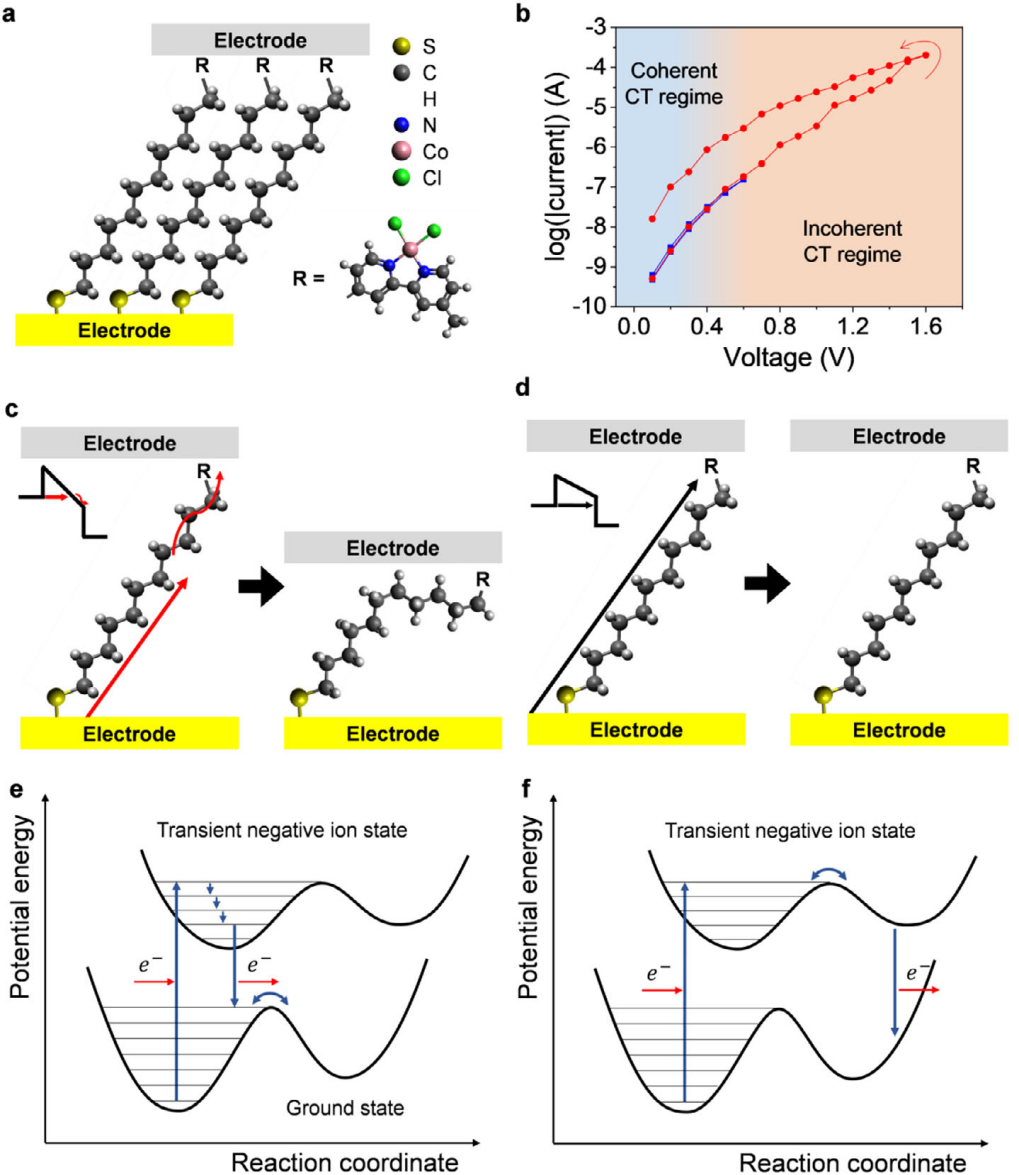
Geometry, molecular design, electrical characterization, and mechanism of conductance hysteresis. a) Schematic representation of the molecular tunneling junction composed of SAMs of *2*,*2*′‐bipyridine‐terminated alkanethiolates: Au^TS^‐S(CH_2_)_11_BIPY‐Co(II)Cl_2_//Ga_2_O_3_/EGaIn. b) Tunneling current in log‐scale versus applied voltage traces measured in BIPY‐CoCl_2_ junctions when 0 V → 1.6 V → 0 V voltage sweep is applied (red dots) and when 0 V → 0.6 V → 0 V voltage sweep is applied (blue dots). c,d) Schematic illustrations of the mechanisms of electron transport across the molecular junctions: (c) Incoherent CT induces the conformational changes of the SAMs, (d) while coherent CT does not. e,f) Schematic illustration of the potential energy surfaces for C─C bond rotation induced by charge injection to the LUMO of the alkyl chain. Red arrows indicate the movement of an electron, and blue arrows indicate changes in vibrational states. e) The C─C bonds can potentially rotate in the TNI state. f) If the reaction does not occur in the TNI state, the molecule returns to the vibrationally excited ground potential energy surface, reducing the energy barrier for C─C bond rotation.

We fabricated a molecular junction using SAMs of alkanethiolates terminated with *2*,*2*′#x02010;bipyridine (BIPY)‐cobalt chloride chelates on a template‐stripped gold substrate, with EGaIn serving as the top electrode (**Figure** **1**a). The sulfur groups of the molecules form covalent bonds with the gold electrode, while the EGaIn electrode establishes a noncovalent contact with the bipyridyl chelates. We applied a voltage to the EGaIn electrode while grounding the gold electrode and measured the electrical properties of the molecular junctions. In a previous study, Park et al. investigated the relationship between the charge transport (CT) mechanism and conductance hysteresis in BIPY junctions complexed with transition metals.^[^ ^7^ ^]^ The junctions exhibited conductance hysteresis only when the voltage entered the incoherent CT regime. Park et al. hypothesized that this hysteretic behavior arises from electron‐orbital interactions when electrons flow through the LUMO of the molecule (the incoherent CT).

Figure 1b illustrates the differences in the *I‐*‐*V* curves when sweeping the applied voltage up to 0.6 V (blue) and 1.6 V (red). The *I‐*‐*V* curves show hysteretic behavior when sweeping the voltage to 1.6 V, while no hysteresis is observed in the range of 0.6 V. Compared to the forward scan (0–1.6 V), the reverse scan (1.6–0 V) exhibits a higher current through the molecular junctions. As reported,^[^ ^7^ ^]^ the molecular junctions begin to show conductance hysteresis when the mechanism of charge transport shifts from coherent CT to incoherent CT (Figure S1, Supporting Information). The transition to the incoherent tunneling regime can be determined from the Fowler‐Nordheim (FN) plot by observing the linear dependence of ln(J V^−2^) on 1 V^−1^. FN plot with a voltage range up to 0.4 V does not indicate a linear dependence, suggesting the molecular junction remains in the coherent CT (Direct tunneling) regime (Figure S2a, Supporting Information).^[^ ^8^ ^]^ In contrast, the FN plot with a voltage range up to 0.6 V shows a linear dependence of ln(J V^−2^) on 1 V^−1^ (Figure S2b, Supporting Information), indicating a transition from coherent CT (direct tunneling) to incoherent CT (FN tunneling and transport through LUMO) between 0.4 and 0.6 V.

The positive correlation between the charge transport mechanism and hysteresis in BIPY‐CoCl_2_ junctions suggests that electron injection into the LUMO of the alkyl chain—the incoherent CT—induces the conductance hysteresis.^[^ ^7^ ^]^ Given those junctions complexed with copper chloride (instead of cobalt chloride) do not exhibit conductance hysteresis (Figure S3, Supporting Information), we exclude the other possibilities originated from the organometallic complex (e.g., rotation,^[^ ^9^ ^]^ and halide anions^[^ ^10^ ^]^) and the electrodes (e.g., reduction/oxidation^[^ ^11^ ^]^ and trapping/de‐trapping process^[^ ^12^ ^]^) because both junctions complexed with CoCl_2_ and CuCl_2_ have fixed conformations and the same core structure. There are two possible causes of conductance hysteresis: i) changes in the molecular state associated with reduction/oxidation reactions during charge transport^[^ ^2^, ^13^ ^]^ and ii) a decrease in the thickness of the tunneling barrier due to molecular conformational changes.

For the changes in the molecular oxidation state, the oxidation state of the molecule may change as charge transports, potentially altering its charge transport characteristics. To determine whether the observed conductance hysteresis originates from a change in the molecular oxidation state, we performed cyclic voltammetry (CV) measurements on SAMs composed of BIPY‐CoCl_2_ and BIPY‐CuCl_2_ complexes. Analysis of the cyclic voltammograms (Figure S4, Supporting Information) within the voltage range of −0.2–1.0 V reveals that the cobalt complex does not exhibit redox peaks, whereas the copper complex displays reversible anodic (E_pa_ = 260 mV) and cathodic (E_pc_ = 600 mV) peaks. The half‐wave potential (*E*_1/2, *NHE*_) referenced to the normal hydrogen electrode (NHE) is equivalent to *E*_1/2, *Ag*/*AgCl*(3*M* *KCl*)_ + 0.197 V, which can also be expressed as (*E_pa_*  +  *E_pc_*)/2  +  0.197 V. The highest occupied molecular orbital (HOMO) energy relative to the vacuum level is determined by *E_HOMO_* = *E*_*abs*,*NHE*_  − *eE*_1/2, *NHE*_, where *E*_*abs*,*NHE*_ =   − 4.5 eV. Applying this relationship yields an E_HOMO_ value of ≈−5.13 eV for BIPY‐CoCl_2_. Considering the Fermi level of the Au/EGaIn electrode (≈4.3 eV), the HOMO of the copper complex becomes accessible within a −1 V bias, meaning the BIPY‐CuCl_2_ complex can undergo oxidation and reduction within the ±1 V bias window. However, the BIPY‐CuCl_2_ junction does not exhibit conductance hysteresis within this ±1 V bias window, whereas the BIPY‐CoCl_2_ junction does exhibit conductance hysteresis, which shows no redox peaks. Based on this observation, we rule out the possibility that conductance hysteresis arises from oxidation/reduction reactions of the molecule.

In the incoherent CT regime, conformational changes may occur due to interactions between electrons and the molecule, potentially leading to conductance hysteresis. Results obtained from confocal Raman spectroscopy at 0 and 1 V reveal differences in Raman peaks (Figure S5, Supporting Information), indicating changes in molecular conformation when voltage is applied in the incoherent CT regime. These structural changes in the molecule are presumed to result from the rotation of a C─C single bond, which experiences comparatively lower steric hindrance than the BIPY moiety. The hysteretic behavior is attributed to changes in the conformation of the alkyl chain,^[^ ^4^, ^14^ ^]^ induced by electron injection. In the incoherent CT regime, electrons tunnel to the LUMO of the molecule and flow through the LUMO (Figure 1c, left). The injected electrons excite the molecule to higher energy states. This increased energy enables the molecules to overcome the energy barrier for C─C bond rotation, resulting in the formation of *gauche* conformations (Figure 1c, right). In the coherent CT regime, electrons tunnel through the entire molecule without inducing conformational changes (Figure 1d). The conformational changes in SAMs, induced by interactions between tunneling electrons and the LUMO of the molecule, reduce the overall thickness of the SAMs, thereby increasing the tunneling current.

To investigate the changes in molecular height within the incoherent CT regime, we performed in situ height measurements using conductive‐atomic force microscopy (C‐AFM). As the AFM cantilever approaches the sample surface, a transient adhesion force arises between the tip and the surface, briefly causing the tip to adhere to the sample and inducing a sudden change in cantilever deflection. This change can be quantified by measuring the difference in the deflection. Using conductive AFM mode, we lowered the cantilever onto the surface of BIPY‐CoCl_2_ SAMs under various bias conditions (0, 0.2, 0.4, 0.6, 0.8, and 1.0 V). While the deflection remained relatively unchanged from 0 to 0.4 V, it began to increase at voltages above 0.6 V (Figure S6, Supporting Information), which supports the interpretation. When the tip is brought into contact under a bias corresponding to the coherent CT regime, the molecular length remains unchanged. In the incoherent CT regime, meanwhile, the molecular length decreased upon contact, resulting in a larger deflection difference.

We presume potential energy surfaces for the incoherent CT‐induced conformational changes of the alkyl backbone in BIPY‐CoCl_2_ molecules, as follows. In the incoherent CT regime, electrons from the electrode tunnel into the LUMO of the molecule, causing the molecule to partially transition to a transient negative ion (TNI) state once the electrons occupy the LUMO.^[^ ^15^ ^]^ The injected electrons excite the molecule to a higher vibrational state within the TNI potential energy surface, thereby lowering the potential energy barrier for C─C bond rotation. When the electron exits the LUMO and transfers to the opposite electrode, the molecule reverts to the ground (neutral) potential energy surface. Two reaction pathways for the conformational changes are possible. First, the reaction may occur within the TNI potential energy surface if the molecule has sufficient energy and time to overcome the potential energy barrier between all‐*trans* and *gauche* conformations in the TNI state (Figure 1e). Second, if the reaction does not proceed within the TNI potential energy surface, the molecule decays back to a vibrationally excited state on the ground potential energy surface (Figure 1f). In both the TNI state and ground state, the vibrational excitation effectively lowers the potential energy barrier and facilitates C─C bond rotation.^[^ ^16^ ^]^ The rotational barrier for C─C bonds in isolated alkane is known to be ≈16–19 kJ mol^−1^.^[^ ^3^ ^]^ According to density functional theory (DFT) calculations, all C─C bond rotational barriers in BIPY‐CoCl_2_ exhibit similar values (Figure S7, Supporting Information). In SAMs, steric interaction between molecules may further increase the rotational barrier. We do not know how many electrons are involved in the rotation. A continuous flow of electrons from the electrode may cumulatively transfer energy to the molecules, enabling structural changes in the molecule.

**Figure 2:**
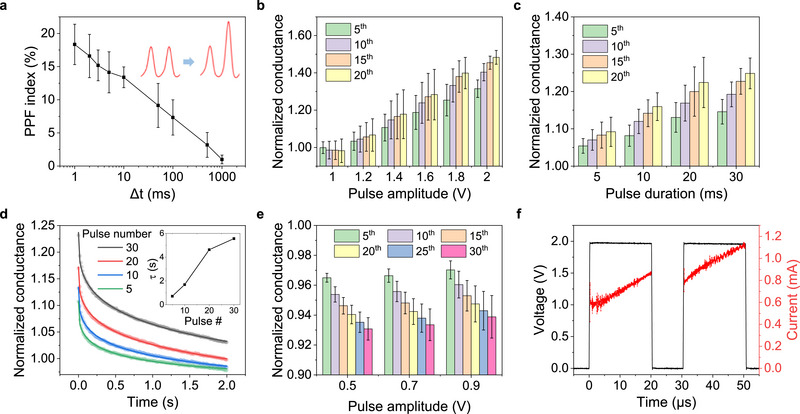
Synaptic behaviors emulated by BIPY‐CoCl_2_ junctions. a) PPF index versus time interval (Δ*t*) in log scale between two pulses. The inset illustrates PPF behavior observed in a biological synapse. Error bars represent the standard deviations of 7 to 13 experiments. Variations in normalized conductance as a function of b) amplitude and c) duration of the potentiation pulse for different numbers of pulses. Error bars represent the standard deviations of 5 to 12 experiments. d) Decay curves for normalized conductance after different numbers of potentiation pulses are applied. The inset shows how the extended relaxation time constant varies with the number of applied potentiation pulses. e) Variations in normalized conductance versus the amplitude of depression pulse for different numbers of pulses. Error bars represent the standard deviations of 4 experiments. f) Conductance enhancement observed with paired pulses of 2 V with a pulse width of 20 µs.

The BIPY‐CoCl_2_ junctions can be viewed as analogous to a biological synapse. In this analogy, the presynaptic and postsynaptic neurons correspond to the bottom Au electrode and top EGaIn electrode, respectively, with the synapse emulated by the BIPY‐CoCl_2_ SAMs. The conductance of the BIPY‐CoCl_2_ junctions represents the synaptic weight, and the variable conductance in response to electrical stimulus emulates synaptic plasticity. Paired‐pulse facilitation (PPF) is a key feature of short‐term synaptic plasticity, where the postsynaptic current is enhanced by a pair of presynaptic stimuli.^[^ ^17^ ^]^ **Figure** **2**a illustrates the PPF behavior in the BIPY‐CoCl_2_ junctions with a pair of pulses (1.4 V, 30 ms; where voltage and time denote the amplitude and the width of the applied pulse, respectively) as a function of various time intervals (Δ*t*) between the pulses (Figure S8a, Supporting Information). The enhancement of the postsynaptic current (PPF index) is calculated using the following equation:

(1) $$\mathrm{PPF}\;=\frac{I_{2}-I_{1}}{I_{1}}\;\times 100\%$$

where *I*_1_ and *I*_2_ represent the current amplitudes of the first and second pulses, respectively. The PPF index decreases exponentially as the time interval increases (Figure S8b, Supporting Information). At the shortest interval (Δ*t* = 1 ms), ≈18% conductance enhancement (PPF index = 18), whereas at the longest interval (Δ*t* = 1 s), the postsynaptic current remains nearly unchanged (PPF index = 0.99). The PPF behavior originates from the interplay between the electric field‐driven conformational changes during the pulse within the incoherent CT regime and the autonomous structural relaxation during the interval. The dependence of PPF behavior on the time interval can be fitted using a stretched exponential function (Figure S8b, Supporting Information).

(2) $$\mathrm{PPF}=A\exp\left[-{\left(\frac{\Delta t}{\tau }\right)}^{\beta }\right]$$

where τ and β correspond to the relaxation time constant and the stretch index (0 < β < 1), respectively. In the BIPY‐CoCl_2_ junctions, τ and β are respectively 61 ms and 0.34, which align well with the time constants observed in biological synapses.^[^ ^18^ ^]^ The stretched exponential fitting suggests that some molecules undergoing structural changes experience a delayed recovery within the ensemble.

Figure 2b illustrates the dependence of molecular conductance on pulse amplitude. We applied 20 consecutive pulses with varying amplitudes (1.0–2.0 V) and a fixed duration (10 ms), measuring the conductance at each pulse by applying a read pulse (1.0 V, 10 ms). The junctions do not show a change in conductance with 1.0 V pulses even after the 20 pulses, because 1.0 V was used as the read pulse. A noticeable enhancement in conductance, however, was observed as the pulse amplitude increased. A voltage higher than the read pulse (1 V) induces a more pronounced conformational change. Accordingly, conductance gradually increased with the number of applied pulses, and the magnitude of conductance enhancement grew with increasing pulse amplitude. As the pulse amplitude in the incoherent CT regime increases, electrons with higher potential energy can flow through a longer region of the orbital, increasing the electron lifetime—the duration an electron resides in the LUMO. The tunneling probability in the molecular junctions also increases with the applied bias. The increase in potential energy and electron lifetime likely enables more conformational changes in the TNI state. The heightened tunneling probability leads to more electrons participating in charge injection, resulting in greater conformational changes both in the TNI state and the ground state.

We investigated the effect of pulse duration on conductance using a similar approach. We applied 20 pulses with a fixed amplitude of 1.4 V but varied durations (5, 10, 20, and 30 ms). Figure 2c shows that the conductance increases more significantly with longer pulse durations. The ratio of the change in molecular conductance after 20 pulses increased from 9% to 24% as the pulse duration increased. A longer pulse duration in the incoherent CT regime allows more electrons to participate in intramolecular charge transport, leading to more electron‐orbital interactions in the BIPY‐CoCl_2_ junctions.

Figures S9 and S10 (Supporting Information) demonstrate that the conductance of BIPY‐CoCl_2_ junctions increases with the number of applied pulses. Once the external electric field is removed, the increased conductance gradually returns to its initial level through spontaneous structural relaxation. This behavior is similar to the phenomenon observed in biological synapses, where the synaptic strength increases with repetitive signal processing (learning) and decreases over time in the absence of further stimulation (forgetting). To investigate the forgetting process, we measured the conductance decay without electrical stimulation after increasing the conductance with different numbers of pulses (5, 10, 20, 30) in the incoherent CT regime (1.4 V, 30 ms). Conductance was recorded using short‐read pulses (1.0 V, 1 ms) every 10 ms (Figure 2d). The forgetting curve was fitted using the Kohlrausch law equation,^[^ ^19^ ^]^ which can be expressed in a form similar to the stretched exponential function used in the previous PPF curve fitting.

For a better comparison of the forgetting curves, we used the averaged *β* values from the four curves.^[^ ^19a,b^ ^]^ The inset of Figure 2d shows that the relaxation time constant increases from 0.71 s to 5.56 s with the increasing number of applied pulses. This mimics the process of short‐term memory converting to long‐term memory through repeated stimuli. The greater the conformational change in SAMs, the longer it takes for them to recover to their initial state.^[^ ^20^ ^]^ The long relaxation time on the order of seconds is attributed to the relaxation of the liquid metal (the EGaIn electrode) in forming a noncovalent contact with SAMs.^[^ ^21^ ^]^ We hypothesize that the changes in the SAMs structure lead to alternations at the SAMs//EGaIn interface, causing a slower reorganization of the liquid metal to form a new interface in response to the SAMs’ changing structure. The rate of this reorganization is significantly slower than the rate of the structural relaxation of the SAMs. This long time constant, on the order of seconds, is also observed in the PPF behavior, where no enhancement in conductance is seen when the time interval between pulses is 1 s (Figure 2a).

Figure 2e illustrates the change in conductance when 30 pulses of varying amplitudes (0.5, 0.7, and 0.9 V) with a pulse width of 20 ms are applied after elevating the conductance with 30 potentiation pulses (1.4 V, 30 ms). Despite being in the incoherent CT regime, the conductance gradually decreases (Figure S11, Supporting Information). We believe this decrease is due to the relaxation process dominating the effect of charge injection. As previously noted, tunneling probability increases with higher applied bias. When fewer electrons with lower potential energy (0.5–0.9 V) are injected compared to the potentiation pulses (1.4 V), these lower‐voltage pulses are insufficient to maintain the deformed molecular structure. However, as the voltage increases within the 0.5–0.9 V range, it more effectively suppresses relaxation. The normalized conductance slightly increases with higher applied bias, implying that higher pulse amplitudes suppress the relaxation process.

For a qualitative evaluation of the minimum energy required for conductance changes, we applied much shorter pulses to emulate PPF behavior. Specifically, pulses with a short duration of 20 µs and an amplitude of 2 V were applied to the BIPY‐CoCl₂ junctions, and the resulting conductance enhancement was observed (Figure 2f). The conductance of BIPY‐CoCl₂ junctions can be modulated with low energy consumption compared to other devices. The energy required to change conductance was calculated using the equation E = IVt, where E is the energy, I is the average current (707 µA) during the pulse, V is the magnitude of the pulse (2V) and t is the pulse duration (20 µs). The obtained energy value was then normalized by the contact area of the EGaIn electrode. As a result, the energy required to change conductance was determined to be 8.0 pJ·µm^−2^. Considering the contact area (3500 µm^−2^) and the effective contact ratio (≈10^−4^) of the EGaIn electrode,^[^ ^22^ ^]^ and the surface coverage (5.7 ×  10^−10^ mol cm^−2^) of BIPY‐Co junctions,^[^ ^23^ ^]^ the energy required to modulate conductance per molecule is calculated to be 23.3 fJ·molecule^−1^. The energy consumption of 8.0 pJ·µm⁻^2^ represents an ≈113‐fold improvement in energy efficiency compared to conventional CMOS‐based devices.^[^ ^24^ ^]^ Furthermore, when evaluated at the molecular level, the energy efficiency of this device is comparable to that of highly efficient 2D material‐based ultra‐low‐power devices.^[^ ^2a,b^ ^]^

Memristor crossbar arrays serve as neural network accelerators.^[^ ^25^ ^]^ In neural networks, the connections—described by weights—between one layer of *n* neurons and another layer of *m* neurons can be represented by a weight matrix *W* of dimensions *m* × *n*. When an input vector *x* of dimensions *n* × 1 is applied to this layer, the output vector *y* is calculated by multiplying *y*  =  *W* · *x*. The resulting output vector *y* is a column vector with dimensions *m* × 1, and this process is known as matrix‐vector multiplication (MVM). In a memristor crossbar array, this MVM can be performed in a single operation (Figure S12, Supporting Information).^[^ ^26^ ^]^ When an input voltage vector, proportional to the input vector, is applied through the word lines, current flows in each cell proportional to the product of the applied voltage and the conductance. According to Kirchhoff's law, the current at each bit line can be represented as ∑*V_i_G*_*i*,*j*_, which corresponds to the output vector. The parallel structure of the memristor crossbar array allows it to perform multiple dot products in a single operation, enhancing operation efficiency during the MVM process.

**Figure 3:**
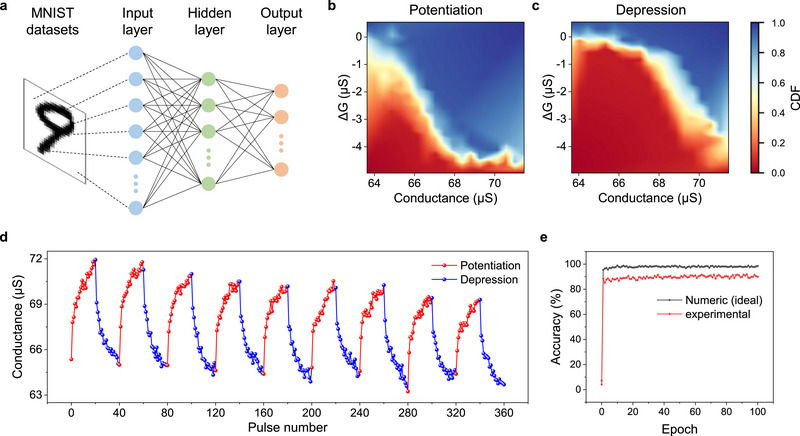
MNIST pattern recognition simulation. a) Schematic diagram of the neural network crossbar simulator with 784 input layers, 300 hidden layers, and 10 output layers. Look‐up table showing conductance variation (Δ*G*) versus conductance for b) potentiation and c) depression. d) Potentiation/depression behavior of BIPY‐CoCl_2_ junctions over 9 cycles. Each cycle consists of 20 pulses of potentiation and 20 pulses of depression. e) MNIST image recognition accuracy achieved using BIPY‐CoCl_2_ junctions compared to an ideal numeric case.

In a memristor array, the conductance matrix represents the weight matrix. To explore the weight‐update properties of BIPY‐CoCl_2_ junctions, we used a set of training pulses consisting of 20 potentiation pulses (1.4 V, 30 ms) and 20 depression pulses (0.1 V, 15 ms). The junctions exhibit gradual and reversible potentiation/depression behavior over nine cycles of training (**Figure** **3**d). The linearity, symmetry, and cycle‐to‐cycle variation of the weight‐update behavior are critical factors affecting the learning accuracy of the memristor crossbar array as a neural network accelerator.^[^ ^27^ ^]^ We tested the weight‐update behavior in the BIPY‐CoCl_2_ junctions for pattern recognition. Using the CrossSim platform, we simulated pattern recognition for MNIST handwritten digit datasets with a three‐layer perceptron neural network (input, hidden, and output layers). The neural network used in the simulation comprised 784 neurons in the input layer, 300 neurons in the hidden layer, and 10 neurons in the output layer (Figure 3a). The 784 input neurons correspond to the values of each pixel in the 28 × 28 pixel MNIST images, and the 10 output neurons correspond to the classification of the 10 digits (0 to 9). The conductance‐update behavior of reversible potentiation/depression cycles (Figure 3d) was used to create lookup tables (LUTs). LUTs provide information on the probability of conductance change (Δ*G*) at a given conductance value (*G*), as illustrated in the cumulative density function (CDF) graphs in Figure 3b,c. The weights were trained using the backpropagation algorithm with 60 000 images, and the learning results were verified using 10 000 test images. After 50 epochs of training using the BIPY‐CoCl_2_ crossbar array, the recognition accuracy reached ≈90% (Figure 3e).

**Figure 4:**
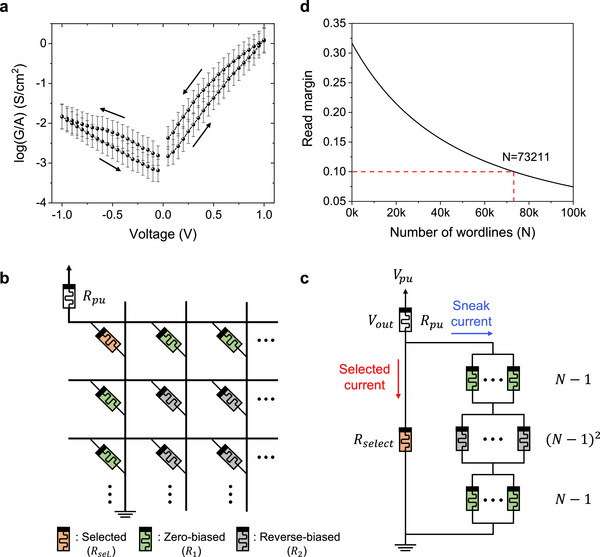
Rectifying properties of BIPY‐CoCl_2_ junctions. a) *I‐*‐*V* curve of BIPY‐CoCl_2_ junctions demonstrating rectifying behavior. Error bars represent the standard deviations of 609 experiments. b) Schematic illustration of an *N* × *N* crossbar array circuit. The modified half‐bias scheme divides the cells into selected cells, zero‐biased cells, and reverse‐biased cells when a bias is applied. c) Equivalent circuit diagram of the crossbar array using the One‐BLPU method. d) Calculated read margin as a function of the number of wordlines, using the half‐bias scheme.

BIPY‐CoCl_2_ junctions not only have the capability to adjust conductance but also exhibit rectifying properties that current flow at positive voltages is higher than at negative voltages.^[^ ^28^ ^]^ **Figure** **4**a shows the *G‐*‐*V* curve measured with a bidirectional voltage sweep (−1.0–1.0 V) for the BIPY‐CoCl_2_ junctions, confirming that charge transport driven by charge injection in alkyl chains exhibits both conductance hysteresis and rectification. The conformational changes of the molecule, which are not fully recovered during the reverse scan in the positive bias regime, gradually restore across the negative bias region. This recovery process results in a decrease in conductance in the negative bias region. To evaluate the durability of BIPY‐CoCl₂ junctions, we conducted continuous *G–V* sweeps over 100 cycles. The junctions maintained stable conductance and rectification properties without significant degradation, demonstrating their robustness for long‐term operation (Figure S13, Supporting Information). The rectification ratio (*G* ^+^/*G* ^−^) in the BIPY‐CoCl_2_ junctions is ≈80. This rectifying characteristic is advantageous because it suppresses sneak currents in a crossbar array, thereby eliminating the need for selectors in high‐density, high‐integration crossbar arrays. Ideally, current flows through the selected cell and exits via the selected bit line when voltage is applied to the selected word line. In a real device, however, sneak currents flow through other word lines and bit lines connected to the selected cell. If each resistive device exhibits rectifying properties, sneak currents can be suppressed, as less current flows through cells other than the selected one.

To evaluate the effectiveness of rectifying properties of the BIPY‐CoCl_2_ junctions in suppressing sneak currents, we adopted a one‐bit line pull‐up (One‐BLPU) approach to simplify the circuit model of the crossbar array (Figure 4b).^[^ ^29^ ^]^ Assuming a *N*×*N* crossbar array, we calculated the read margin using the *I*‐*V* characteristics of the molecules and a modified half‐bias scheme. We considered the worst‐case scenario where all unselected cells are in the low resistance state (LRS) to maximize the sneak current. Under this scenario, the crossbar array can be divided into three regions: the selected cells (for *R*_*sel*._), region 1 (*R*_1_), and region 2 (*R*_2_). Region 1 and region 2 consist of (*N*‐1) cells sharing either a bit line or a wordline with the selected cell, and (*N*‐1)^2^ cells that do not share both the bitline and wordline with the selected cell, respectively. With the modified half‐bias scheme (Figure S14, Supporting Information), *V_read_* is applied only to the selected cell, ≈0 V is applied to the cells in region 1, and − *V_read_* is applied to the cells in region 2 (Figure 4b).^[^ ^30^ ^]^ The sneak current flowing through the crossbar array can be represented as an equivalent circuit (Figure 4c). Using One‐BLPU, the pull‐up resistor (*R_pu_*) is connected to the selected bitline while the selected wordline is grounded, causing all the cells except the selected cell to be floated. When the pull‐up voltage (*V_pu_*) is applied to the selected bitline, the voltage across the pull‐up resistor (*V_out_*) allows us to distinguish between the LRS and high resistance state (HRS) states of the selected cell. A higher Δ*V_out_*, the difference in *V_out_* when the selected cell is in HRS versus LRS, is desirable. The read margin, Δ*V_out_*/*V_pu_*, is calculated using Kirchhoff's equation,

(3) $$R_{sneak}=\frac{{\left.R_{1}^{LRS}\right|}_{0.01\;V}}{N-1}+\frac{{\left.R_{2}^{LRS}\right|}_{-V_{read}}}{{\left(N-1\right)}^{2}}+\frac{{\left.R_{1}^{LRS}\right|}_{0.01\;V}}{N-1}$$

(4) $$R_{crossbar}^{HRS}=R_{sel.}^{HRS}\;∥R_{sneak}$$

(5) $$R_{crossbar}^{LRS}=R_{sel.}^{LRS}\;∥R_{sneak}$$

(6) $$\mathrm{Read}\;\mathrm{margin}\;=\frac{\Delta V_{out}}{V_{pu}}=\left(\frac{R_{pu}}{R_{crossbar}^{LRS}+R_{pu}}-\frac{R_{pu}}{R_{crossbar}^{HRS}+R_{pu}}\right)$$

We determined the value of *R_pu_* as the resistance of the LRS selected cell in the crossbar ($R_{sel.}^{LRS}$) to maximize Δ*V_out_*.^[^ ^31^ ^]^ Each resistance was derived from the *I‐*‐*V* curve of BIPY‐CoCl_2_ junctions and the resistance at nearly 0 V was obtained using the resistance at 0.01 V (Figure S15, Supporting Information). Figure 4d presents the calculated read margin values as a function of array size *N*. A read margin of 0.1 is considered the minimum criterion for determining the maximum array size.^[^ ^29^, ^32^ ^]^ In the worst‐case scenario, the BIPY‐CoCl_2_ junctions can support a maximum array size of *N* = 73211, indicating they possess rectifying properties that effectively suppress sneak currents and allow for operations in a 73211  ×  73211 crossbar array without the need for additional selectors (Figure 4d).

## 3 Conclusion

Using molecular junctions based on Au^TS^‐S(CH_2_)_11_BIPY‐CoCl_2_//Ga_2_O_3_/EGaIn, we successfully demonstrated a synaptic device, which emulates various synaptic functions. The device features a simple two‐terminal structure, and we can easily control its conductance by modulating the applied voltage, which governs the mechanism of charge transport. We hypothesize that electrons injected into the LUMO of the molecules via incoherent CT excite the molecules into a vibrationally excited TNI state, which subsequently decays to the vibrationally excited ground state. In both potential energy surfaces, the added vibrational energy enables the molecules to undergo conformational changes. This modulation of conformational states in the SAMs allows the BIPY‐CoCl_2_ junctions to emulate phenomena such as PPF, SADP, and SDDP while achieving molecular conductance changes with low energy consumption of 23.3 fJ·molecule^−1^. The reversible potentiation/depression behavior was used to simulate neural networks, achieving an accuracy of 90% in MNIST pattern recognition. BIPY‐CoCl_2_ junctions exhibit both memristive and rectifying behaviors simultaneously. Calculations of the read margin indicate that the bifunctionality of BIPY‐CoCl_2_ junctions could potentially suppress sneak currents when fabricated into a crossbar array up to the size of 73211 × 73211. Despite these promising characteristics, precise quantitative control remains challenging due to the high number of rotational sites within the molecule and the relatively low rotational barrier. To enhance the stability and reproducibility of conductance modulation, future studies should focus on tuning the rotational barrier to achieve improved control over molecular conformational states. Additionally, while efforts to integrate molecular devices into large‐scale architectures are progressing, significant challenges remain in ensuring their reliability, scalability, and compatibility with existing fabrication techniques. Addressing these issues will be crucial for advancing molecular electronics toward practical applications. Compared to established technologies associated with solid‐state memristors, the phenomenon of changing molecular conductance through charge injection that we report may offer unique characteristics. By inputting information into small molecules via pulses with limited rotational motion at room temperature, our approach presents a potential method for long‐term information storage with minimal additional energy consumption or information loss, addressing some of the limitations of solid‐state electronics.

## Conflict of Interest

The authors declare no conflict of interest.

## Author Contributions

J.P. and Y.L. conceived and designed the overall experiment. J.P. supervised the whole project. C.L. and T.K. led the project and drafted the initial manuscript. C.L., T.K., and Y.P. conducted the electrical characterization of the junction. T.K. performed the DFT calculations. C.L. took the lead in Crossim simulations. D.K., C.S., Y.P., T.K., and J.P. carried out the AFM experiments. C.L., T.K., and J.P. analyzed both the experimental and computational results. C.L., T.K., J.‐L. L., Y.L., and J.P. revised the manuscript based on the discussions with all authors and discussed the proposed mechanistic pathway. All authors contributed to the review and editing of the manuscript.

## Supporting information



Supporting Information

## Data Availability

The data that support the findings of this study are available from the corresponding author upon reasonable request.
