# Supplementary material for: Electric Field‐Driven Conformational Changes in Molecular Memristor and Synaptic Behavior
Source: Adv Sci (Weinh). 2025 Apr 30;12(23):2505016. doi: 10.1002/advs.202505016 (PMC12199394; doi:10.1002/advs.202505016)
Supplement: Supplementary file 1 — Supporting Information [file ADVS-12-2505016-s001.docx]

**Supporting Information for:**

**Electric Field-driven Conformational Changes in Molecular Memristor and Synaptic Behavior**

**­­­­­****­­­­­**Chanjin Lim,^1,⊥^ Taegil Kim,^1,⊥^ YoungJu Park,^1^ Daeho Kim,^2^ ChaeHo Shin,^3^ Suji Ha,^1^ Jin-Liang Lin,^4^ Yuan Li,^4,*^ and Junwoo Park,^1,5,*^

^1^ Department of Chemistry, Sogang University, Seoul 04107, Republic of Korea

^2^ Bruker Nano Surface, Bruker Korea Co, Ltd., Seoul 05840, Republic of Korea

^3^ Division of Chemical and Material Metrology, Korea Research Institute of Standards and Science, Daejeon 34113, Republic of Korea

^4^ Key Laboratory of Organic Optoelectronics and Molecular Engineering, Department of Chemistry, Tsinghua University, Beijing 100084, China

^5^ Center for Nano Materials, Sogang University, Seoul 04107, Republic of Korea

⊥ C.L. and T.K. contributed equally to this work.

**Experimental Section**

*Materials and Methods*

We followed previously reported procedures for the preparation of materials and experimental methods,^[25a]^ and included a description of the experiments that were conducted in the same way.

*Materials*

All reagents were used as supplied unless otherwise specified. All organic solvents in analytical grade (99%) were purchased from Sigma-Aldrich. High purity eutectic gallium-indium (EGaIn; 99.99%) was obtained from Sigma-Aldrich and used as supplied.

*Choice of Electrodes*

Molecules (*i.e.*, S(CH_2_)_11_BIPY) were deposited on spontaneously aligned on the template-stripped Au surfaces (Au­­^TS^).^[1]^ The roughness of bottom electrode plays critical role on the electrical properties of SAMs, particularly rough surfaces decrease the packing order or induce the defects in SAMs. To minimize the defects of the gold surfaces, we used the ultraflat Au­­^TS^ substrate. Eutectic gallium indium (EGaIn), a liquid metal alloy, is used as the top electrode which enables the non-damaging contact with SAMs. Au­­^TS^/SAMs//GaO_x_/EGaIn junction enables the measurement under ambient conditions.^[1b]^

*Preparation of BIPY-CoCl_2_ junctions*

Our synthesis of the BIPY-containing SAMs, which comprised of BIPY moiety-terminated alkanethiolates, followed by the literature.^[25b]^ SAMs of S(CH_2_)_11_BIPY were formed by immersing the templated-stripped Au substrate into the 1.0 mM ethanolic solutions of thiol-terminated molecules overnight under a nitrogen atmosphere. After immersion, the samples are gently rinsed with ethanol for 1 minute, and dried under slow flow of nitrogen gas. Prepared Au­­^TS^-S(CH_2_)_11_BIPY were again immersed into the 10.0 mM ethanolic solution of Cobalt(II) chloride overnight for the chelation of BIPY moiety with CoCl_2_. After rinsing and drying as previously done, BIPY-CoCl_2_ junctions were prepared.

*Characterization: I-V measurements*

All the measurements of the samples were done on the anti-vibration table. Electrical measurements were done with the semiconductor characterization system (Keithly 4200A-SCS). We connected the negative port to the grounded Au surface, and the positive port of a source meter to a microliter syringe (Hamilton®, HAM80075) containing EGaIn alloy, served as a top electrode. We formed a conical shaped EGaIn tip by extruding an EGaIn droplet on a clean Si wafer.^[20]^ The soft and delicate contact between SAMs and the conical-shaped EGaIn is controlled with the micromanipulator. We applied the voltage to the EGaIn tip and measured the current flowing across the molecular junctions.

*In-situ conductive-atomic force spectroscopy (C-AFM)*

The surface morphology and height variations of BIPY-CoCl₂ SAMs were analyzed using an atomic force microscope (Dimension Icon, Bruker). For conductive atomic force microscopy (C-AFM) measurements, a conductive probe (DDESP-V2, Bruker) with a nominal probe radius of 100 nm and a probe spring constant of 80 N/m was employed. During the measurements, a voltage was applied to the tip while approaching the sample surface. The deflection-height curve was extracted before and after the onset of adhesion.

*Confocal Raman spectroscopy*

To investigate the impact of applied potential on the molecular conformations of BIPY-CoCl₂ junctions, we utilized confocal Raman spectroscopy to analyze the Raman spectra of self-assembled monolayers (SAMs). The Raman measurements were conducted using a Horiba LabRam HR Evolution confocal Raman microscope with a 633 nm excitation source (~10 mW). Each spectrum was acquired with a 150 µm pinhole and a 600 gr/mm grating, averaging five measurements per spot, each with an acquisition time of 300 s. In total, spectra were collected from 25 different spots on the SAMs.

Prior to each measurement, the spectrograph was calibrated using a Si wafer reference (520.7 cm⁻¹ peak), and stray cosmic rays were removed from the spectra. Background subtraction was performed using a reference spectrum from a bare Au sample. The Raman measurements were taken through the Au layer, with samples prepared on a 30 nm template-stripped Au substrate and encapsulated with an optical adhesive. The applied voltage was controlled via a Keithley 2400 source meter, using an EGaIn electrode to interface with the SAM.

*Density-functional theory calculations*

Quantum chemical calculations were performed at the density functional theory (DFT) level using ORCA 5.0.0 software^[2]^ with the B3LYP functional. Geometry optimizations and subsequent calculations were conducted using the def2-SVP basis set for all atoms. To determine the rotational barrier associated with C–C bond rotation, a surface scan was performed by varying the dihedral angle in 10° increments.

*Artificial neural network simulation*

The CrossSim platform was used to perform the verify the pattern recognition for the Modified National Institute of Standard and Technology (MNIST) handwritten digit datasets. The simulation for the pattern recognition was conducted with a three-layer perceptron neutral network. The neural network consists of 784 input neurons, 512 hidden neurons, and 10 output neurons. A look-up table, derived from nine repeated potentiation–depression cycles in a single junction, served as the basis for the simulation. For training, 60,000 images were used to update the synaptic weights using backpropagation algorithm. Subsequently, the pattern recognition accuracy was tested with 10,000 test images.

**Figure S1.** Plot of the magnitude of conductance hysteresis versus applied voltage window. The magnitude of conductance is defined as the maximum value of G_rev_/G_fwd_. G_fwd_ and G_rev_ are Conductance of forward and reverse sweep. Hysteresis of conductance starts to occur near 0.5 V, which is the transition voltage of BIPY-CoCl_2_ junctions.

\

**Figure S2.** Fowler-Nordheim (FN) plots for junctions in BIPY-CoCl_2_ junctions. (a) FN plot of 0.4 V bias window does not show the transition, meaning the direct tunneling dominates the charge transport. (b) FN plot of 0.6 V bias window shows the transition, indicating the FN tunneling occur at high bias region.

**Figure S3.** Averaged *J*(*V*) traces of Au^TS^-S(CH_2_)_11_BIPY-MCl_2_//GaO_x_/EGaIn junctions. (a) M = Co and (b) M = Cu.

**Figure S4.** Cyclic voltammograms of SAMs of S(CH)_11_BIPY-CuCl_2_ and S(CH)_11_BIPY-CoCl_2_. The experiments were conducted in aqueous 0.1 M KClO_4_ solutions, utilizing an Ag/AgCl reference electrode immersed in 1.0 M KCl (aq) and a Pt counter electrode. The BIPY-CuCl_2_ SAMs exhibited reversible anodic peaks (E_pa_ ≈ 260 mV) and cathodic peaks (E_pc_ ≈ 600 mV). From these values, the HOMO level of S(CH)_11_BIPY-CuCl_2_ was calculated to be −5.13 eV.

**Figure S5.** Confocal Raman spectrum of BIPY-CoCl_2_ at 0 V and 1 V after excitation at 633 nm. Peaks near 990 cm⁻¹ are attributed to ring-breathing modes, those around 1450 cm⁻¹ correspond to CH deformation modes, and peaks at approximately 1300 cm⁻¹ arise from in-plane ring vibrations.^[3]^

**Figure S6.** A plot showing the deflection differences(Δd) observed when approaching the sample with varying tip bias applied through C-AFM.

**Figure S7.** DFT calculation results for an alkane chain based on the dihedral angle. The dihedral angles were determined using the labeled carbon number as the primary axis.

**Figure S8.** Paired-pulse facilitation (PPF) behavior in BIPY-CoCl_2_ junctions. (a) The PPF behavior in the BIPY-CoCl_2_ junction. (b) PPF index versus time interval (Δ*t*) in linear scale between two pulses and the fitted curve. The curve is fitted with stretched exponential decay function.

**Figure S9.** Change in the normalized conductance after applying 20 potentiation pulses with different amplitude.

**Figure S10.** Change in the normalized conductance after applying 20 potentiation pulses with different duration.

**Figure S11.** Change in the normalized conductance after applying depression pulses with different amplitude. 30 potentiation pulses with 1.4 V amplitude and 30 ms width are applied before the depression pulses.

**Figure S12.** Schematic illustration of the matrix-vector multiplication (MVM) operation in a memristor crossbar array.

**Figure S13.** (a) The G-V curve measured for 100 cycles in BIPY-CoCl_2_ junction. (b) high Conductance state (on) at -0.1 V and low conductance state (off) measured over 100 cycles.

**Figure S14.** Schematic illustration of the modified half-bias scheme in the crossbar array. The red cell is the selected cell where *V_read_* is applied. The other cells, including the green cells where 0 V is applied and the gray cells where −*V_read_* is applied, are sneak cells.

**Figure S15.** The *I-V* curve of BIPY-CoCl_2_ junctions showing rectifying properties.

**References**

[1] a) E. A. Weiss, G. K. Kaufman, J. K. Kriebel, Z. Li, R. Schalek, G. M. Whitesides, *Langmuir* **2007**, *23*, 9686-9694; b) R. C. Chiechi, E. A. Weiss, M. D. Dickey, G. M. Whitesides, *Angew. Chem.-Int. Edit.* **2008**, *47*, 142-144.

[2] H. J. Yoon, K.-C. Liao, M. R. Lockett, S. W. Kwok, M. Baghbanzadeh, G. M. Whitesides, *J. Am. Chem. Soc.* **2014**, *136*, 17155-17162.

[3] F. Neese, *WIREs Comput. Mol. Sci.* **2022**, *12, e1606*.

[4] X. Yan, P. Li, L. Yang, J. Liu, *Analyst* **2016**, *141*, 5189-5194.
